# Feasibility, safety and efficacy of multi-dose vagus nerve stimulation in Parkinson’s disease: a double-blind, randomised sham-controlled proof-of-concept study

**DOI:** 10.1007/s00415-025-13430-4

**Published:** 2025-10-09

**Authors:** Hilmar P. Sigurdsson, Heather Hunter, Lisa Alcock, Evangeline E. Maughan, Harvey Bramley, Philip Brown, Giovanni Palermo, Mark R. Baker, John-Paul Taylor, Lynn Rochester, Alison J. Yarnall

**Affiliations:** 1 Translational and Clinical Research Institute, Faculty of Medical Sciences, Newcastle University, Newcastle upon Tyne, UK; 2 National Institute for Health and Care Research (NIHR) Newcastle Biomedical Research Centre (BRC), Newcastle University, Newcastle Upon Tyne, UK; 3 The Newcastle Upon Tyne Hospitals NHS Foundation Trust, Newcastle upon Tyne, UK; 4 Unit of Neurology, Department of Clinical and Experimental Medicine, University of Pisa, Pisa, Italy

**Keywords:** Randomised controlled trial, Parkinson’s disease, Vagus nerve stimulation, Feasibility, Gait, Attention

## Abstract

**Background:**

Gait and cognitive impairments are hallmark features of Parkinson's disease (PD) that significantly impact quality of life. These deficits arise from both dopaminergic and non-dopaminergic mechanisms, including cholinergic dysfunction. Transcutaneous cervical vagus nerve stimulation (tcVNS) is a non-invasive neuromodulatory technique that may enhance cholinergic function and has potential therapeutic relevance for individuals with PD. However, its feasibility, acceptability, adherence, and safety in a domiciliary setting remain unclear. We conducted a single-centre, double-blind, parallel, sham-controlled, randomised proof-of-concept study.

**Objectives:**

The primary objective was to assess the feasibility, acceptability, adherence, and safety of home-based tcVNS. The secondary objective was to explore preliminary effects on gait and cognitive function.

**Methods:**

Thirty-three participants with PD were randomised to either active (*n* = 16) or sham (*n* = 17) tcVNS and self-administered two stimulations twice daily for 12 weeks, followed by a 24-week post-intervention follow-up.

**Results:**

Retention was high (93.9%), with two participants withdrawing from the sham group for reasons unrelated to the intervention. No serious adverse events were reported during the intervention period. Mild adverse events led to discontinuation of tcVNS use in three active and two sham participants. tcVNS had minimal effects on gait and cognition, although small, non-significant improvements in step length variability were observed.

**Conclusions:**

This study represents the longest tcVNS intervention trial in PD to date and supports the feasibility, acceptability, and safety of domiciliary tcVNS. While preliminary findings suggest tcVNS may have therapeutic potential, larger trials are needed to establish its effectiveness for improving gait and cognitive function in PD.

**Supplementary Information:**

The online version contains supplementary material available at 10.1007/s00415-025-13430-4.

## Introduction

Parkinson’s disease (PD) is a complex, multi-system movement disorder characterised by the progressive loss of dopamine-producing neurons in the midbrain, which affects both central and peripheral nervous systems [1]. PD is the fastest-growing neurological condition globally [2], presenting a constellation of motor (e.g. tremor, bradykinesia) and non-motor symptoms (e.g. cognition, dysautonomia) that significantly impair quality of life. Among the former, gait impairments are hallmark motor symptoms that contribute substantially to patient disability and increase the risk of falls and reduced mobility [3]. Addressing gait impairment is consistently prioritised by patients as a critical focus of research because it has a profound impact on independence and well-being [4, 5].

Levodopa is the primary treatment for PD, effectively improving bradykinesia and rigidity [6]. However, its impact on gait and posture is limited [7] and specific gait characteristics progressively decline even if patients are treated with traditional dopaminergic therapies [8].

In addition to motor deficits, cognitive impairment is prevalent in PD, affecting various cognitive domains, including attention, memory, and executive function [9, 10]. This, in addition to the progression of gait impairments which appear independent of dopaminergic treatment, suggests the involvement of non-dopaminergic and alternative neuropathological mechanisms.

Cholinergic dysfunction is a likely contributor. In PD, cholinergic denervation occurs early, with significant loss of cholinergic neurons in the nucleus basalis of Meynert, which provides extensive projections to the cerebral cortex [11], and the pedunculopontine nucleus, which projects mainly to the thalamus [12]. Growing evidence suggests that dysfunction of nbM negatively impacts cognitive function, whereas atrophy of the PPN is associated with gait disorders in PD [13–16]. These findings help strengthen the case for targeting the cholinergic system as an adjunctive therapeutic strategy to improve gait and cognitive outcomes in PD.

Vagus nerve stimulation is an emerging neuromodulation technique that applies electrical impulses to the vagus nerve via either the auricular (taVNS) branch [17] or the cervical (tcVNS) branch using a handheld device [18]. Collectively, these two approaches are termed non-invasive (transcutaneous) vagus nerve stimulation (tVNS). Acetylcholine is the primary neurotransmitter of the vagus nerve and plays a crucial role in modulating central and peripheral nervous systems. tVNS is thought to activate cholinergic receptors, which may have widespread therapeutic effects, including reducing inflammation and enhancing neuroplasticity via the cholinergic anti-inflammatory pathway [19–21]. This has led to an increasing interest in the potential of tVNS as an adjunctive treatment for autoimmune and neurodegenerative conditions. This includes Sjögren's syndrome [22], stroke rehabilitation to restore lost motor function [23], and PD [24, 25]. Given its dual impact on central and peripheral pathways, tVNS may be particularly relevant for addressing motor and non-motor symptoms in PD.

An earlier study from our own group investigated the effects of a tcVNS on gait in people with PD. In this randomised, sham-controlled trial, 30 participants with PD were allocated to either active or sham tcVNS groups. Gait was the primary outcome measure and was assessed before and after a single dose of tcVNS. The analysis focused on discrete gait characteristics, specifically step length variability and step time variability, which are adversely affected in PD, are resistant to dopaminergic replacement therapies, and may be underpinned by cholinergic neurotransmission [26–29]. The findings from this study showed that both step length variability and step time variability improved in participants receiving the active stimulation compared to those receiving sham stimulation, with changes in step length variability reaching statistical significance [30].

Based on these results, which provide preliminary evidence supporting the role of tcVNS in modulating key gait characteristics that are otherwise difficult to treat in PD, we set out to assess if tcVNS is feasible in people with PD when stimulation is delivered repeatedly at home over a 12-week period. We sought to determine if this type of intervention is acceptable in PD, whether participants adhere to the protocol, and if tcVNS is safe as a treatment. Evaluating the feasibility of domiciliary tcVNS is important for understanding if this intervention can be realistically implemented in real-world settings, where adherence and usability are paramount for long-term success.

The primary objectives of this study were to develop and administer the study protocol, establish feasibility indicators, and assess the acceptability, adherence, and safety of multi-dose tcVNS in PD. We describe the intervention as *multi-dose* to distinguish it from our previous single-dose study, as participants in the present study self-administered multiple (two; twice a day) stimulations per day (as described in “Intervention*”*). We wanted to determine the potential therapeutic effects of tcVNS, focusing on gait and cognitive function as proof-of-concept measures. Our analyses centred on discrete gait characteristics including the variability of step length and step time, hypothesising that tcVNS would improve (reduce) step length and step time variability. In addition, we assessed changes in cognitive domains of attention, executive function, and visual memory to provide a broader perspective on the intervention’s potential benefits, with the hypothesis that active tcVNS would improve these cognitive domains as they are likely underpinned by cholinergic neurotransmission [15, 31, 32].

## Materials and methods

A detailed description of the trial design and procedures has been published [33]. Our manuscript has been prepared in full compliance with the Consolidated Standards of Reporting Trials (CONSORT) guidelines [34] and, as appropriate, the guidelines for minimum reporting standards in research on tVNS [35]. This study was prospectively registered (ISRCTN19394828).

### Protocol amendments

Minor protocol amendments were made during the study, including adjustments to eligibility criteria and follow-up visit windows to accommodate participant and clinical scheduling. Full details of these amendments are provided in the Supplementary Materials.

### Trial design

This study was a single-site, proof-of-concept, double-blind, sham-controlled randomised trial with two parallel treatment groups conducted in the UK. Participants with PD were randomly assigned to receive active or sham tcVNS. Both participants and investigators involved in data collection and analysis were blinded to group allocation.

Participants were assessed at three time points: baseline (before the intervention), after 12 weeks of tcVNS use (post-tcVNS), and after a 12-week non-stimulation period (follow-up) for a total study duration of 24 weeks. During the 12-week intervention period, participants self-administered tcVNS as instructed. Then, participants stopped using the device and were re-assessed. The follow-up assessment was conducted to evaluate any potential beneficial carryover effects of tcVNS after the cessation of stimulation. A comprehensive clinical and demographic review was carried out at baseline.

### Participants

This study aimed to establish proof of concept and assess feasibility in preparation for larger clinical trials. A recruitment target of 40 participants was set based on comparable studies [36]. A comprehensive list of inclusion and exclusion criteria is available in our published protocol [33]. Briefly, eligible participants included individuals with PD who were dementia free, on stable medication, able to walk independently for at least 2 min, and without significant cardiovascular conditions. Exclusion criteria encompassed a range of medical and procedural factors, such as severe neurological or orthopaedic conditions affecting gait, specific cardiovascular risks, abnormal anatomy or pain at the treatment site, and contraindications related to tcVNS, including implanted devices or prior tcVNS use.

### Recruitment

Recruitment for this study was conducted over a 2-year period, from January 2022 to January 2024. All study assessments were conducted at the Clinical Ageing Research Unit on the Campus for Ageing and Vitality, Newcastle University, in partnership with the Newcastle upon Tyne NHS Hospitals Foundation Trust. All participants provided written informed consent, and study procedures adhered to the Declaration of Helsinki [37]. Ethical approval was granted by the East Midlands—Derby Research Ethics Committee (reference: 21/EM/0177).

### Intervention

The intervention used in this study was the GammaCore® Sapphire tcVNS device (ElectroCore, LLC). This handheld, rechargeable device has signal-generating electronics and a digital interface. Two stainless steel discs on the device deliver low-voltage electrical stimulation targeting the cervical vagus nerve. Participants self-operated the device, adjusting the stimulation intensity to the highest tolerable, nonpainful level.

An unblinded team member (HH) not involved in data collection or analysis trained each participant in proper placement and usage. The device was positioned on the left side of the neck, anterior to the sternocleidomastoid muscle, lateral to the trachea, and above the carotid artery pulse. At the end of the 12-week intervention period, participants were asked to demonstrate the placement technique to the same unblinded team member. We targeted the left vagus nerve to avoid cardiovascular-related events [38].

Participants self-administered two doses of tcVNS twice daily at home for 12 weeks, with each dose delivering 120 s of stimulation using manufacturer-provided specifications (pulse width: 1 ms, pulse frequency: 25 Hz), totalling 8 min daily. While no tcVNS guidelines exist for PD, recommendations for cluster headache management suggest a maximum of 12 applications per day [18].

Active (CE 571,753) and sham devices supplied by ElectroCore, LLC—the manufacturers of the gammaCore® device—were indistinguishable in appearance, weight, and handling; however, only the active device stimulated the vagus nerve. The sham device delivers a low-frequency (0.1 Hz) biphasic signal that produces a small buzzing sensation, aiding in participant blinding; critically, this signal is insufficient to active the vagus nerve. Participants in the sham group followed the same usage instructions as those in the active group.

### Randomisation process

Participants were randomly allocated to receive either active tcVNS or sham tcVNS by the unblinded team member. We used a covariate-adaptive randomisation procedure with a minimisation method to ensure balanced group allocation [39, 40], considering participants’ age, sex, global cognition score (Montreal Cognitive Assessment [MoCA] [41]), and motor severity as measured by the MDS-UPDRS part III [42].

### Primary outcome measures

In the present manuscript, we report on the primary outcomes of feasibility, acceptability, adherence, and safety of multi-dose tcVNS in PD. These evaluation metrics were measured at pre- and post-tcVNS.

*Feasibility* indicators encompassed consent, eligibility, recruitment, and retention rates, in addition to reasons for exclusion, non-participation, and withdrawals from the study. *Adherence* was assessed using device-recorded usage data and calculated based on the proportion of delivered versus expected stimulation minutes. *Acceptability* was evaluated through completion rate and a modified usability questionnaire assessing participant satisfaction and ease of use. *Safety* was monitored through adverse event reporting throughout the study. Events were classified as possibly related or unrelated to tcVNS based on temporal association and clinical judgement. A full description of outcome definitions and calculation methods is provided in the Supplementary Materials.

### Secondary outcome measures

Secondary outcomes evaluated the effects of tcVNS on gait and cognitive function, with assessments carried out at baseline, post-tcVNS, and follow-up.

#### Gait assessment

Gait was assessed in a controlled laboratory setting during a 2-min continuous walking task clockwise around a 25 m oval circuit at each participant’s preferred speed [29]. Footfalls were recorded on a Zeno instrumented walkway (dimensions: 6.1 × 0.6 m, Protokinetics LLC, Haverton, Pennsylvania, data recorded at 120 Hz) positioned along one of the straight sections of the circuit and analysed using the Protokinetics Movement Analysis Software (PKMAS). A schematic diagram of the circuit is presented in Supplementary Fig. 1. The circuit incorporated two wide, arched turns—one at each end—to avoid abrupt changes in direction (e.g. turns). Gait was assessed in all participants in an ‘on’ levodopa state. Primary gait characteristics included step length variability and step time variability, based on findings from our earlier pilot study [30] and evidence of gait variability impairments in PD [29].

#### Cognitive assessment

Cognitive function of attention, executive function, and visual memory were assessed using composite scores including the power of attention (PoA), fluctuating attention (FA), processing speed (PS), and continuity of attention (CoA) via validated computerised tests at each time point [43]. Executive function was measured using the number of problems solved on first choice (OTS-PSFC), and the mean latency to first choice from the One Touch Stockings of Cambridge tasks (OTS-MLFC), and visual memory was assessed using the Paired Associative Learning-Total Errors (PAL-TEA, adjusted to account for incorrect choices and approximated errors on unattempted problems) from CANTAB. A full description of all cognitive measures is provided in our published protocol [33].

### Statistical analysis

Statistical analyses and data visualisations were performed using MATLAB (R2024a, The MathWorks Inc., Natick, MA, USA) and R statistical software (version 4.4.0, http://www.r-project.org).

Analysis of primary outcomes included all participants approached, informed, consented, recruited, and randomised into the study. Analyses were done on the ‘intention-to-treat’ population including those who withdrew from the study and stopped using the device during the 12-week intervention period. We conducted an exploratory ‘per-protocol’ analysis, excluding participants who withdrew, discontinued the device, or demonstrated non-adherence to the tcVNS dosing schedule.

Differences in demographic and clinical characteristics between groups were analysed using independent sample t-tests for continuous variables. For ordinal variables, we used the Wilcoxon rank sum test, and for categorical variables, we used Chi-square (*χ*^2^) tests.

For our secondary objectives, we analysed gait and cognitive data using mixed-model analyses. This included linear models for continuous variables and generalised models for counts to examine the effects of the intervention over t*ime* (i.e. sessions), as per recommendations [44, 45]. This retains statistical power and is true to the intention-to-treat approach. *Time* interacting with *treatment* (active, sham) was entered as a fixed effect in all models, controlling for baseline values (pre-tcVNS) and levodopa equivalent daily dose (LEDD; [46] due to group differences). Random intercepts for participants were included to account for participant-level variability. Model residuals were evaluated for normality using histograms and Q–Q plots, and the Satterthwaite approximation was applied to adjust the degrees of freedom.

Continuous variables, including variability of step length and step time, and reaction times (PoA, FA, PS and OTS-MLFC) were analysed using linear mixed models in the ‘lme4’ [47] and ‘lmerTest’ [48] packages in R, with unstructured covariance matrix. Changes from baseline (Δpost-tcVNS and Δfollow-up) were entered as outcomes, with Δpost-tcVNS measures and the sham group set as the reference time point and group status, respectively.

For cognitive measures represented as counts, including the CoA score, OTS-PSFC, and PAL-TEA, we employed generalised mixed models with a negative binomial distribution. Since negative counts cannot be modelled, raw values from post-tcVNS and follow-up were entered as outcomes for all variables. These models were fitted using the ‘glmmTMB’ package in R [49] with a loglink function specified.

Estimated marginal means were extracted using the ‘ggeffects’ package in R [50]. Effects are reported as unstandardised B coefficients with 95% confidence intervals. Given the exploratory nature of these analyses and a relatively small sample size, a significance threshold of *p* < 0.05 was used without adjustment for multiple testing.

## Results

**Fig. 1:**
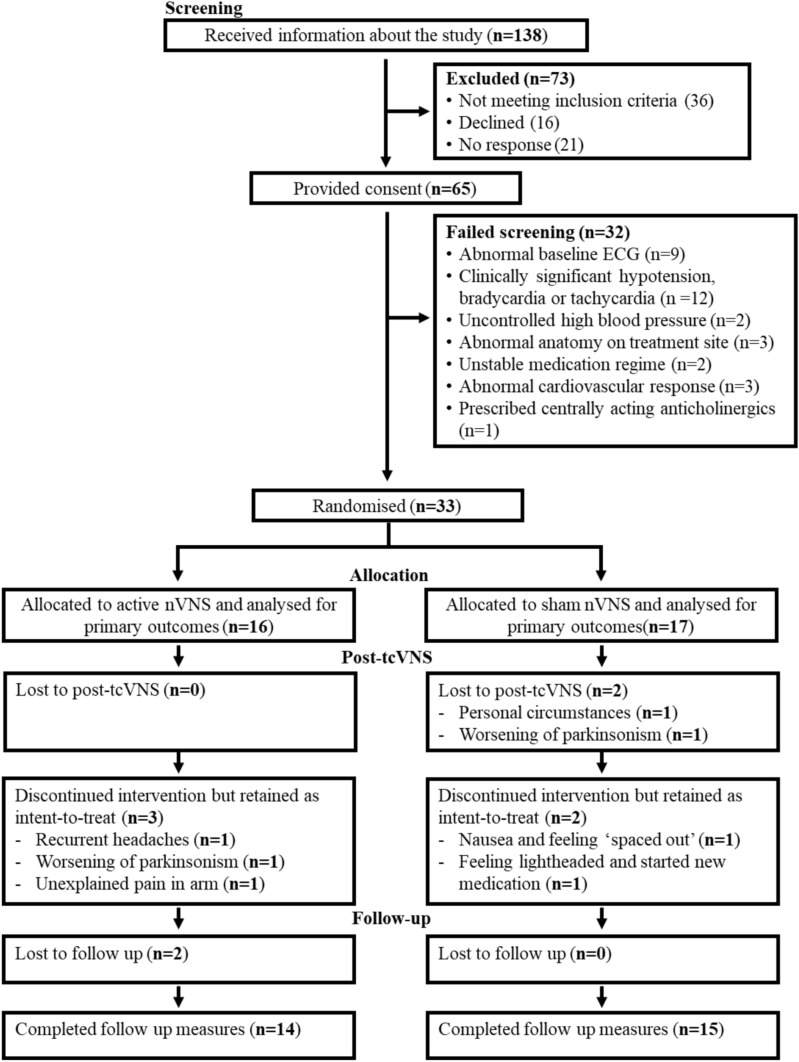
CONSORT diagram of participant flow in the AdVaNSING-PD study

The CONSORT-compliant diagram is depicted in Fig. 1 and summarises the study and participant flow. Based on our randomisation procedure, 16 participants were allocated to the active group and 17 to the sham group. On average, participants were seen at 12.53 weeks (± 0.65) for post-tcVNS and 24.53 (± 0.52) weeks for follow-up assessment from baseline.

### Group characteristics

Table 1 summarises the demographic and clinical characteristics of the participants. Briefly, all participants were White British. Participants in the sham group had significantly higher LEDD (*t*(31) = – 2.99, *p* = 0.005) compared to those in the active tcVNS group. No other demographic or clinical characteristics showed a statistically significant difference between the two groups. Over the 12-week intervention period, two participants had an unanticipated change in their PD medication (1 active group, 1 sham group). For subsequent analyses of the data, one participant discontinued device use and was excluded from the ‘per-protocol’ analyses. The other completed the study and excluding this participant from the ‘per-protocol’ analyses did not alter the results (data not shown).

**Table 1:** Demographic and clinical data measured at baseline

Variable (unit or maximum score)	Active tcVNS	Sham tcVNS	Test statistic	*p*
*N*	16	17		
Age (years)	58.81 ± 8.66	61.28 ± 7.18	*t* = – 0.90	0.38
Sex (M/F)	11 males (68.8%)	12 males (70.6%)	*X*^*2*^ = 0.01	0.91
MoCA (30)	27.75 ± 2.41	27.06 ± 2.61	*t* = 0.79	0.44
GDS-15	3.81 ± 3.54	4.06 ± 3.27	*t* = – 0.21	0.84
Education (years)	11.5 (5)	12 (5)	*W* = – 0.24	0.81
Time from diagnosis (years)	4.51 ± 3.87	7.04 ± 6.23	*t* = – 1.39	0.17
LEDD (mg/day)	461.70 ± 315.69	773.53 ± 283.22	*t* = – 2.99	**0.005**
MDS-UPDRS-III (132)	26.50 ± 10.80	30.59 ± 15.87	*t* = – 0.86	0.40
Faller status			*X*^*2*^ = 1.01	0.60
No falls	12 (75%)	11 (64.7%)		
Single fall	1 (6.3%)	3 (17.65%)		
Recurrent falls	3 (18.7%)	3 (17.65%)		
Resting systolic (mmHg)	129.81 ± 11.54	127 ± 13.03	*t* = 0.53	0.60
Resting diastolic (mmHg)	75.13 ± 7.76	75.13 ± 7.76	*t* = – 0.32	0.75
Resting heart rate (bpm)^a^	73.44 ± 11.12	72.19 ± 9.51	*t* = 0.34	0.73
Standing systolic (mmHg)	125.94 ± 20.16	130.41 ± 14.83	*t* = – 0.73	0.47
Standing diastolic (mmHg)	73.44 ± 8.61	78.24 ± 15.20	*t* = – 1.11	0.28
Standing heart rate (bpm)^a^	78.38 ± 13.33	82.44 ± 21.61	*t* = – 0.64	0.53
Hoehn and Yahr stage, HY (5)			*X*^*2*^ = 3.11	0.21
HY I	0 (0%)	1 (5.88%)		
HY II	16 (100%)	14 (82.35%)		
HY III	0 (0%)	2 (11.76%)		
Clinical Frailty Scale score (9)	3 (0)	3 (1.25)	*W* = – 0.04	0.97

Means are reported for continuous variables. Frequencies with percentages are given for categorical variables. Medians with interquartile ranges are reported for ordinal variables

*M* male, *F* female, *MoCA* Montreal Cognitive Assessment, *GDS-15* Geriatric Depression Scale 15-items, *LEDD* levodopa equivalent daily dose, *MDS-UPDRS-III* Movement Disorder Society Unified Parkinson’s Disease Rating Scale part III, *bpm* beats per minute

Numbers in brackets next to variable names indicate the maximum possible score for that measure or the unit of measurement

^a^Resting and standing heart rate was not recorded for one participant in the sham group

### Evaluating tcVNS as an added treatment in Parkinson’s disease

#### Feasibility of domiciliary multi-dose tcVNS in people with Parkinson’s

**Fig. 2:**
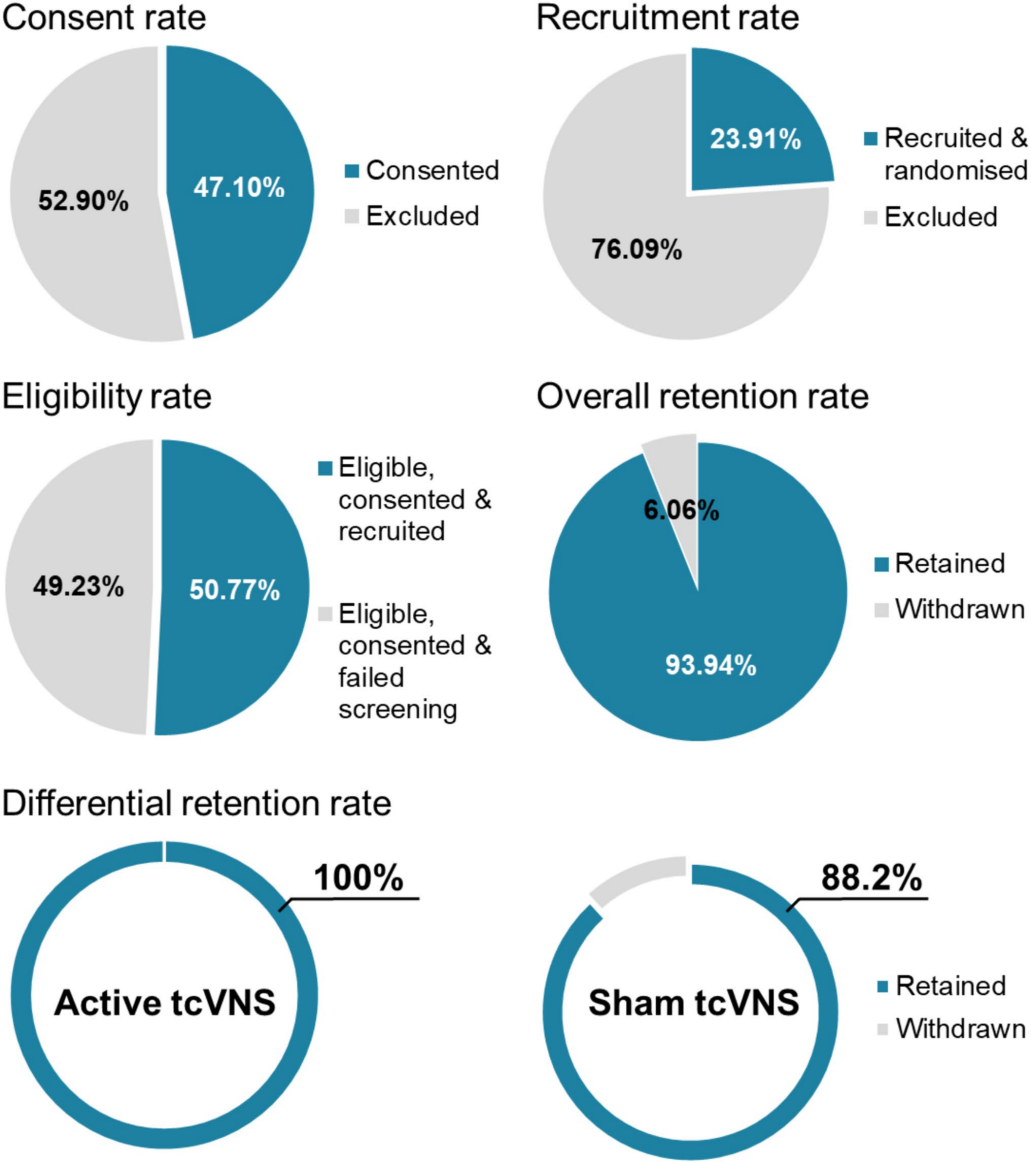
Feasibility metrics of the AdVaNSING-PD study, including consent, eligibility, recruitment, and retention rates

Measures of feasibility are depicted in Fig. 2. Our consent rate was 47.1%. Of the 138 participants initially approached and provided with study information, 73 were excluded: 36 (26.1%) did not meet the inclusion criteria, 16 (11.6%) declined participation, and 21 (15.2%) could not be re-contacted.

The eligibility rate was 50.8%. In total, 65 participants consented to take part in the study, but 32 of these participants failed the screening process in our clinic. The primary reasons for screening failure were cardiovascular issues, including bradycardia, significant hypotension, and abnormal ECG, followed by abnormal anatomy at the treatment site, and medication incompatibility with our inclusion criteria. The recruitment rate was 23.9%, with 33 participants eligible for randomisation out of the 138 individuals who were approached and informed about the study.

During the 12-week intervention period, the overall retention rate was 93.9%. The differential retention rate was 100% for the active tcVNS group and 88.2% for the sham tcVNS group. Two participants in the sham group withdrew during the 12-week intervention period, one due to worsening parkinsonism, and the other for personal reasons. After the 12-week follow-up period, two additional participants withdrew, also for reasons unrelated to the study.

#### Participant adherence to the intervention

**Fig. 3:**
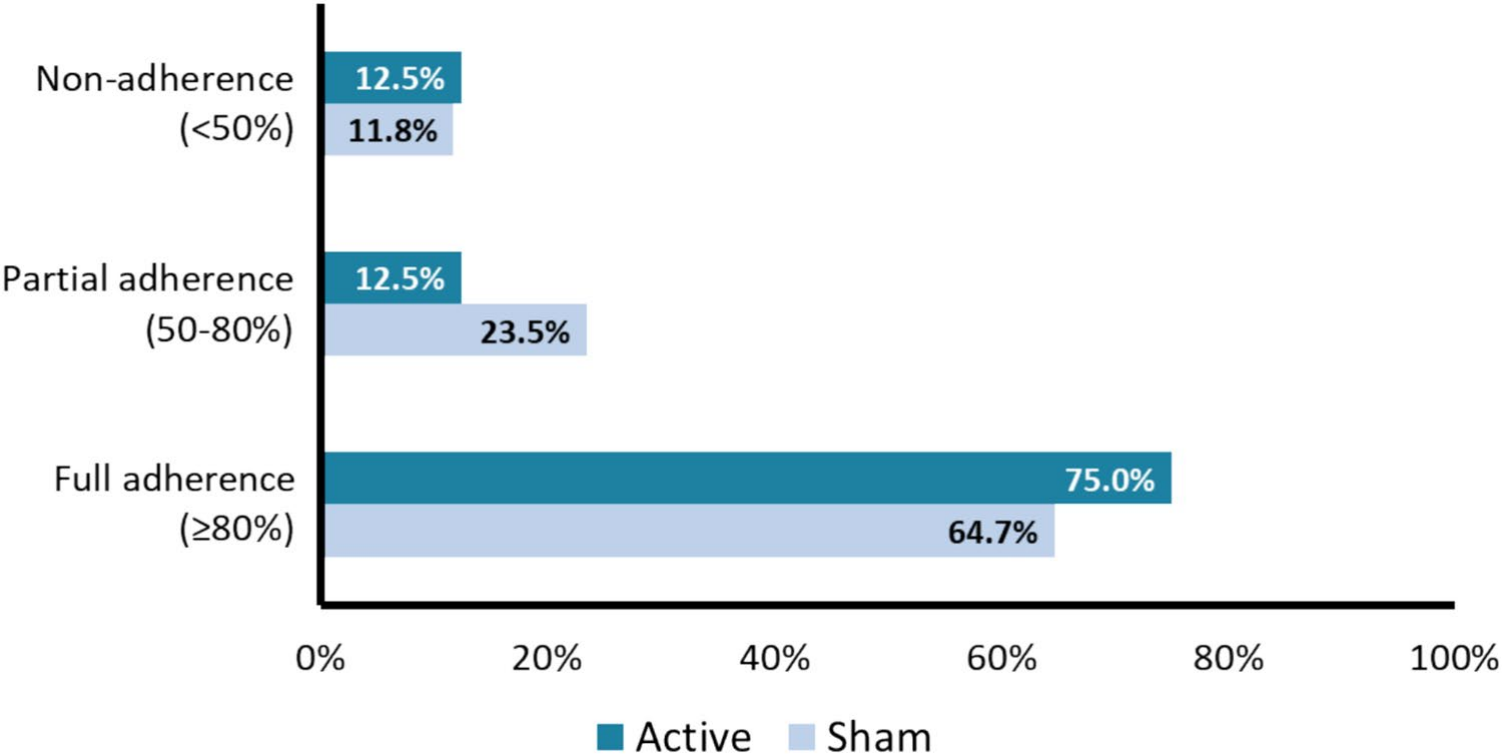
Adherence to the tcVNS intervention in the AdVaNSING-PD study, categorised into full adherence, partial adherence, and non-adherence. Bars represent the proportion of participants in each adherence category for the active and sham groups

While the average stimulation intensity was slightly higher in the active group (23.49 ± 5.68 a.u.) compared to the sham group (20.48 ± 4.90 a.u.), this difference was not statistically significant (*t(33)* = – 1.36, *p* = 0.18). Adherence rates were generally high (Fig. 3), 75% of active participants (*n* = 12) and 64.7% of sham participants (*n* = 11) met the full adherence criteria. Partial adherence was observed in 12.5% (*n* = 2) of the active group and 23.5% (*n* = 4) of the sham group. Non-adherence was comparable between groups (12.5% [*n* = 2] in the active group vs. 11% [*n* = 2] in the sham group). Notably, both non-adherent participants in the sham group had discontinued device use prematurely. The percentage of days with zero usage was slightly lower in the active group (11.9%) compared to the sham group (14.66%).

#### Acceptability of tcVNS in people with Parkinson’s

The overall completion rate of the 12-week intervention period was 78.8%. We also calculated the differential completion rate. In the active group, 18.7% (*n* = 3) of participants discontinued the use of the device, citing reasons which were recorded as possibly related to the use of the device including unexplained headaches (*n* = 1), worsening parkinsonism (*n* = 1), and arm pain (*n* = 1). In the sham group, 11.8% (*n* = 2) discontinued device use due to nausea (*n* = 1) and light-headedness (*n* = 1), which was recorded as possibly related to tcVNS use. All participants remained in the study as intention to treat.

**Fig. 4:**
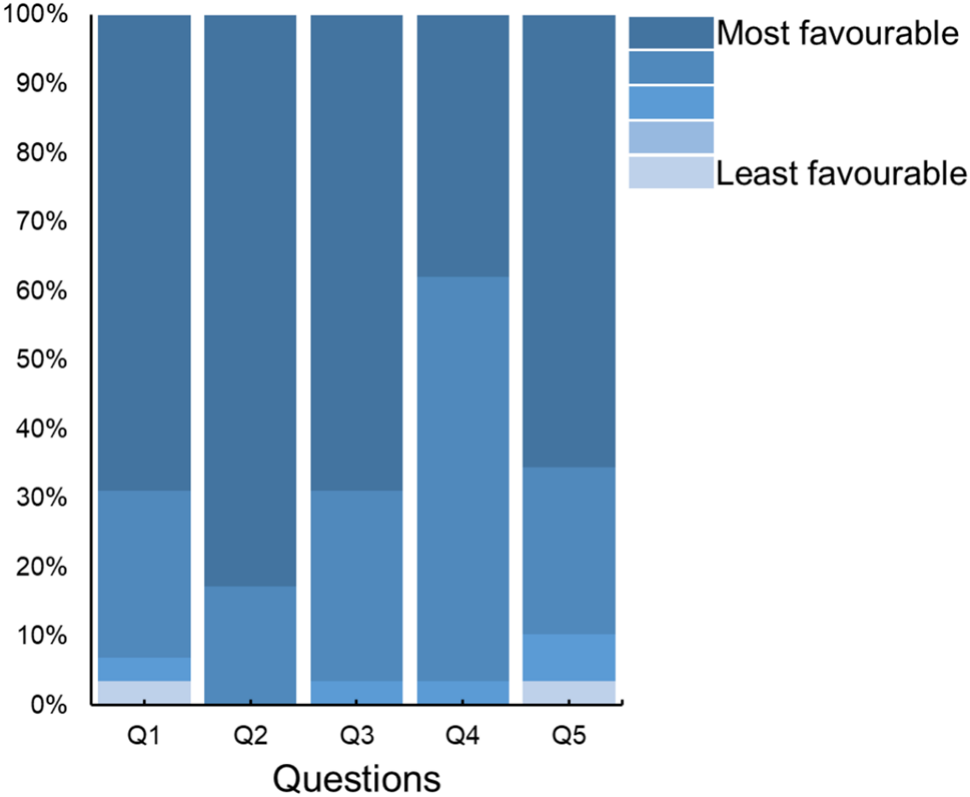
Participant responses to the usability questionnaire as a percentage of total responses. Q1: The instructions on how to use the tcVNS device given to me by the researchers were clear. Q2: It was easy to follow the instructions on how to use the tcVNS device given to me by the researchers. Q3: Using the tcVNS device twice daily was easy. Q4: Using the tcVNS device twice daily was easy to implement into my daily routine. Q5: Using the tcVNS device twice daily interrupted my daily routine

Twenty-nine participants (87.9%) completed the usability questionnaire. Data was missing from two participants, and 2 participants withdrew before the post-tcVNS visit. Overall, there was a general agreement among participants that the instructions by the researchers on how to use the device were clear and easy to follow (see Fig. 4). A large majority of participants selected the most or second most favourable options when asked if using and implementing tcVNS into their daily routine was easy. By contrast, when asked if using tcVNS twice daily interrupted their daily routine, the answers were more spread, suggesting some additional burden.

#### Safety of tcVNS in people with Parkinson’s

During the 12-week treatment period using tcVNS, 49 adverse events were reported (Table 2). This was reported by nine (56.3%) participants in the active group and eight (47.1%) participants in the sham group. No serious adverse events were reported. All adverse events were classed as mild to moderate in severity. Of the 49 adverse events, nine were classified as possibly related to the use of the device. Falls were the most commonly reported adverse event. Although the number of falls was higher in the active group, this was largely driven by one highly active, rural-dwelling participant with prior falls history, and the proportion of participants experiencing falls was lower than in the sham group (active: 12.5%, sham: 17.6%). This pattern may reflect that greater walking activity—even if overall beneficial—can paradoxically increase falls risk, particularly in individuals already predisposed to falling (i.e. with prior fall history) [51]. All falls were classed as being unrelated to tcVNS use.

**Table 2:** Adverse events reported by participants over the 12-week intervention period

	Active tcVNS (n = 16)		Sham tcVNS (n = 17)	
	Number of participants (%)	Number of events	Number of participants (%)	Number of events
Arthritis	0 (0.0%)	0	1 (5.9%)	1
Bruising skin	1 (6.3%)	1	0 (0.0%)	0
Cardiovascular symptoms^a^	1 (6.3%)	1	1 (5.9%)	1
Dental pain and infection^b^	1 (6.3%)	1	1 (5.9%)	1
Dizziness	0 (0.0%)	0	1 (5.9%)	1
Dystonia	1 (6.3%)	1	0 (0.0%)	0
Facial numbness	0 (0.0%)	0	1 (5.9%)	1
Fall	2 (12.5%)	12	3 (17.6%)	5
Gastrointestinal disturbance^c^	0 (0.0%)	0	1 (5.9%)	2
Headache	2 (12.5%)	2	0 (0.0%)	0
Haematuria	1 (6.3%)	1	0 (0.0%)	0
Impulsive behaviours	0 (0.0%)	0	1 (5.9%)	1
Infection^d^	0 (0.0%)	0	2 (11.8%)	2
Injury^e^	1 (6.3%)	1	1 (5.9%)	1
Insomnia	0 (0.0%)	0	1 (5.9%)	1
Lower limb swelling	0 (0.0%)	0	1 (5.9%)	1
Lump on tongue	1 (6.3%)	1	0 (0.0%)	0
Nausea	0 (0.0%)	0	1 (5.9%)	1
Pain^f^	2 (12.5%)	2	0 (0.0%)	0
Parkinsonism^†^	1 (6.3%)	1	1 (5.9%)	1
PD medication change	1 (6.3%)	1	1 (5.9%)	1
Rosacea	1 (6.3%)	1	0 (0.0%)	0
Skin irritation at the stimulation site	1 (6.3%)	1	0 (0.0%)	0
Stiff jaw	0 (0.0%)	0	1 (5.9%)	1

The table presents the number (%) of participants reporting adverse events and the frequency of the adverse events by groups

^†^Parkinsonism refers to the worsening of pre-existing parkinsonism symptoms

^a^Cardiovascular symptoms included increased heart rate recorded on a commercial device (*n* = 1) and orthostatic hypotension (*n* = 1)

^b^Dental pain and infection included toothache (*n* = 1) and tooth abscess (*n* = 1)

^c^Gastrointenstinal disturbance included constipation and gastroenteritis (*n* = 1)

^d^Infection included urinary tract infection (*n* = 1) and viral infection (*n* = 1)

^e^Injury included subluxation distal interphalageal joint 3rd toe (*n* = 1) and broken little toe (*n* = 1)

^f^Pain included a pain in distal arm (*n* = 1) and musculoskeletal pain (*n* = 1)

During the non-stimulation follow-up period (from 12 to 24 weeks), 24 adverse events were reported. The full details of these adverse events are presented in Supplementary Table 1. Five serious adverse events were reported by one participant during hospital admission, all of which were unrelated to the study device (small bowel obstruction, urinary retention, acute kidney injury, infection, and pre-syncope due to medication prescribed for urinary retention). All other adverse events were mild to moderate in severity.

### Effects of tcVNS on dopa-resistant gait characteristics

In all models, visualisations indicated that residuals followed a normal distribution. Raw means and standard deviations are provided in Supplementary Table 2.

**Fig. 5:**
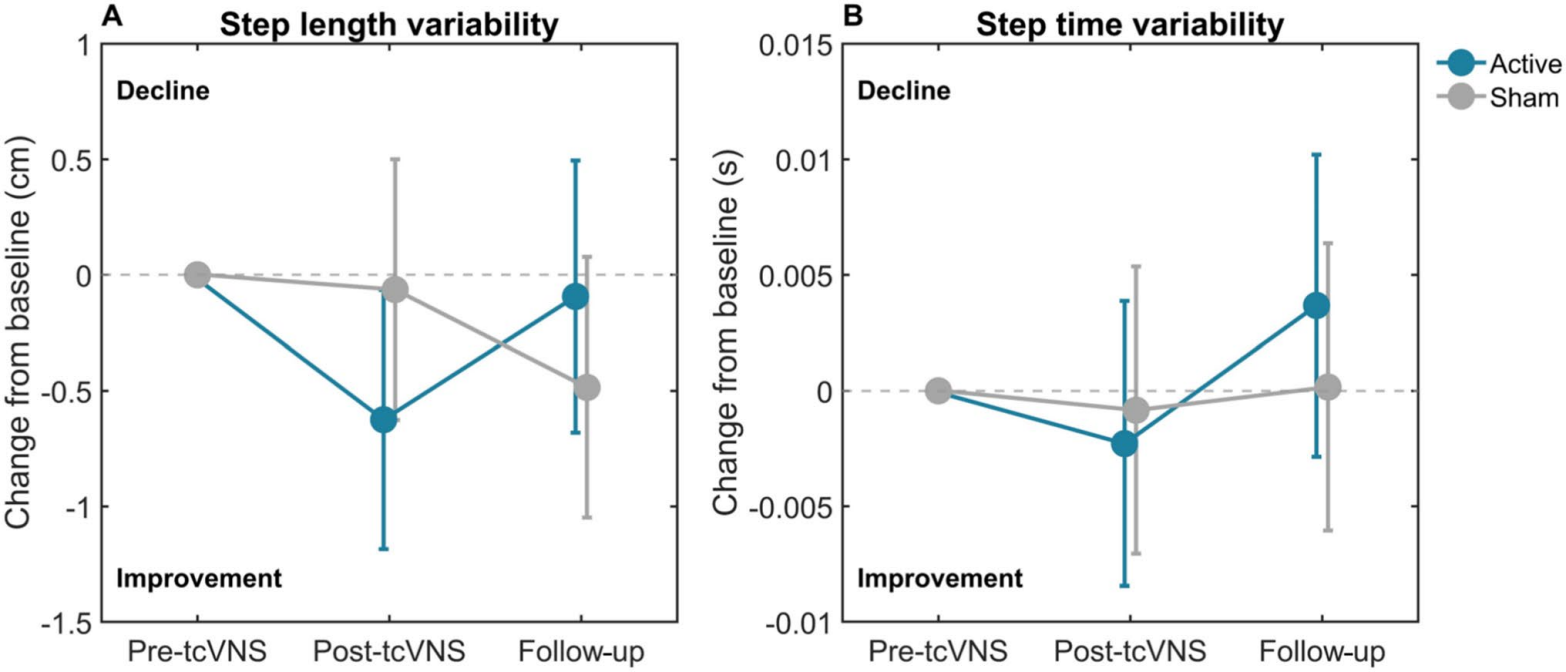
Estimated marginal means (± 95% CI) for the change from baseline (grey dotted line) in discrete gait characteristics for active and sham tcVNS groups. **A** step length variability, **B** step time variability. *cm* centimetres, *s* seconds

Two participants were excluded from this analysis due to a low number of steps captured by the walkway, rendering calculation of gait characteristics unreliable (1 active, 1 sham), leaving 15 in the active group and 16 in the sham group. For step length variability, the linear mixed model revealed no statistically significant effects (Table 3). The main effect of treatment (*B* = – 0.56, *p* = 0.180) indicated a trend towards improvement in the active group (Fig. 5A) post-tcVNS. The treatment × time interaction term showed borderline significance (*B* = 0.95, *p* = 0.053), suggesting a potential difference in the change from post-tcVNS to follow-up between the groups. As seen in Fig. 5A, at 24-week follow-up participants in the active group return to baseline performance. For step time variability, no significant main effects (*B* = – 0.0014, *p* = 0.749) or interaction effects (*B* = 0.0050, *p* = 0.384) were observed (Table 3 and Fig. 5B). Results from the exploratory ‘per-protocol’ analysis showed no significant findings for either gait characteristic analysed (Supplementary Table 3).

**Table 3:** Results from the linear mixed model intention-to-treat analysis comparing active tcVNS with sham tcVNS on the variability of step length and step time

Outcome	Active tcVNS, mean (SD)		Sham tcVNS, mean (SD)		Main effect of treatment (after 12 weeks; post-tcVNS)			Treatment x time interaction (change from post-tcVNS [12 weeks] to follow-up [24 weeks])		
	Δpost-tcVNS	Δfollow-up	Δpost-tcVNS	Δfollow-up	Estimate (95% CI)	SE	*p* value	Estimate (95% CI)	SE	*p* value
Step length variability (cm)	– 0.653 (1.421)	– 0.142 (1.990)	– 0.037 (0.708)	– 0.459 (0.845)	– 0.561 (– 1.39 to 0.27)	0.412	0.180	0.953 (– 0.01 to 1.92)	0.469	0.053
Step time variability (s)	– 0.003 (0.008)	0.002 (0.024)	0.000 (0.004)	0.001 (0.006)	– 0.001 (– 0.01 to 0.008)	0.005	0.749	0.005 (– 0.007 to 0.02)	0.006	0.385

The table shows the average change from baseline to post-tcVNS and follow-up for each group with the corresponding standard deviation, estimates for the main effect of treatment and the treatment x time interaction with standard errors and associated *p* values

### Effects of tcVNS on cognitive function in Parkinson’s disease

**Fig. 6:**
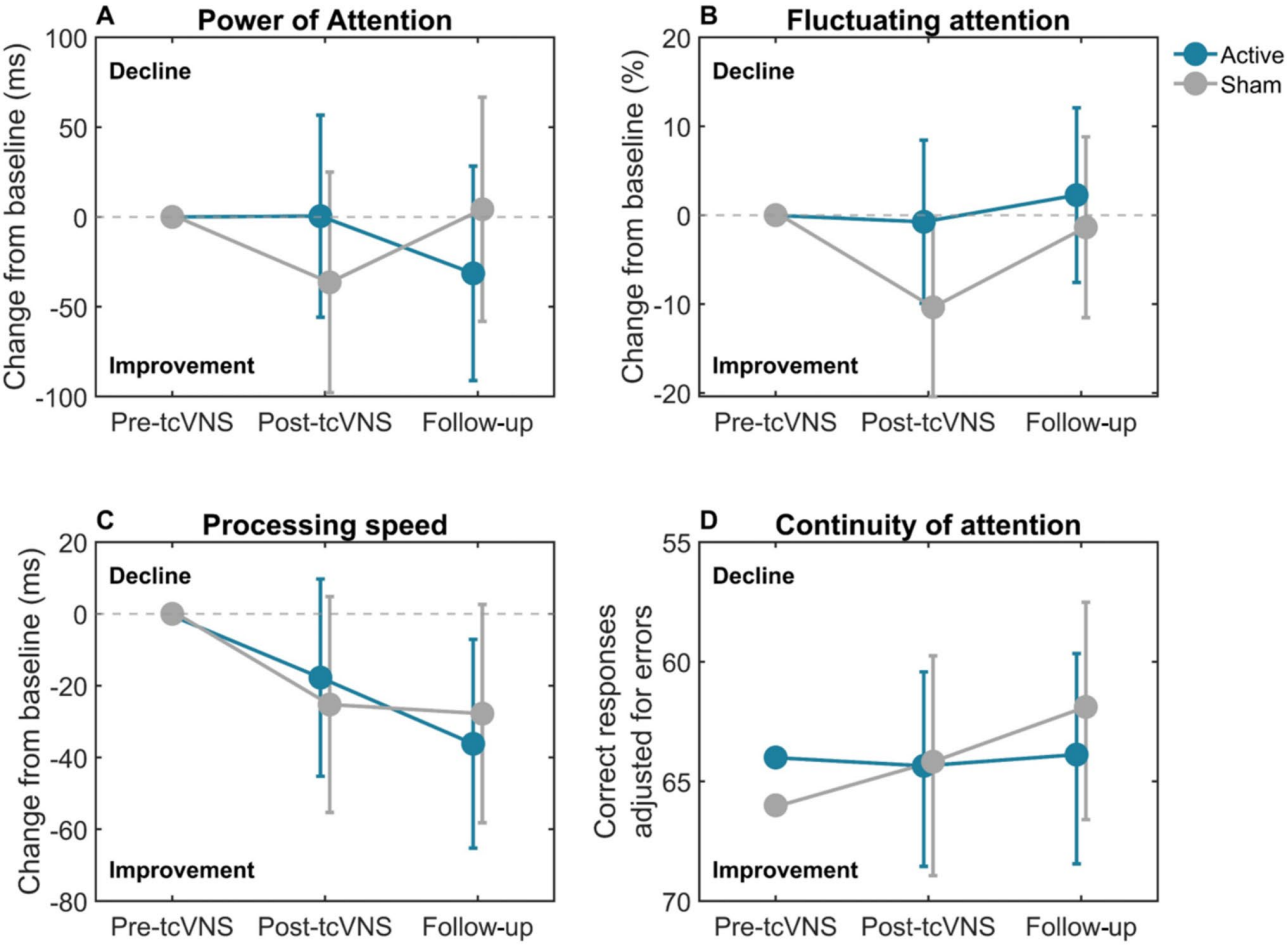
Estimated marginal means (± 95% CI) for the change from baseline (grey dotted line) in the power of attention (**A**), fluctuating attention (**B**), and processing speed (**C**) for Active and Sham tcVNS groups. For Continuity of Attention (**D**) raw rather than change values were analysed. Abbreviations: ms – milliseconds. Note that the CoA plot has been inverted with lower values at the top of the figure for consistency

In all models, visualisations indicated that residuals followed a normal distribution. Estimates for the composite measures of attention are reported in Table 4. For this analysis, two participants were excluded due to measurement errors (sham group), leaving 16 in the active group and 15 in the sham group. For all composite measures (PoA, FA, PS and CoA), the mixed models revealed no statistically significant effects of treatment (Fig. 6). For PoA (Fig. 6A) there was a trend towards a significant treatment × time interaction (*B* = – 72.53, p = 0.1). Looking at the estimated marginal means, while the active group showed no improvement post-tcVNS, they did improve at follow-up, whereas the sham group performed at baseline level. Results from the exploratory ‘per-protocol’ analysis showed no significant effects of tcVNS on any composite measure of attention (Supplementary Table 4).

**Table 4:** Results from the linear mixed model intention-to-treat analysis comparing active tcVNS with sham tcVNS on the cognitive function of attention

Outcome	Active tcVNS, mean (SD)		Sham tcVNS, mean (SD)		Main effect of treatment (at 12 weeks; post-tcVNS)			Treatment × time interaction (change from post-tcVNS [12 weeks] to follow-up [24 weeks])		
	Δpost-tcVNS	Δfollow-up	Δpost-tcVNS	Δfollow-up	Estimate (95% CI)	SE	*p* value	Estimate	SE	*p* value
PoA (ms)^a^	15.38 (140.01)	– 11.75 (115.15)	– 55.90 (98.37)	– 13.78 (107.46)	36.86 (– 50.55 to 124.26)	43.26	0.40	– 72.53 (– 162.6 to 17.54)	43.86	0.11
FA (%)^a^	– 4.50 (18.34)	– 1.43 (33.12)	– 5.95 (9.95)	3.00 (22.87)	9.62 (– 4.88 to 24.11)	7.19	0.19	– 5.99 (– 22.20 to 10.22)	8.07	0.46
PS (ms)^a^	– 24.46 (55.99)	– 39.95 (58.55)	– 18.23 (47.64)	– 21.27 (74.02)	7.48 (– 36.36 to 51.32)	21.71	0.732	– 15.88 (– 60.08 to 28.32)	21.54	0.47
CoA (n)^b^	1 (4)	1.5 (4)	0 (3)	– 2 (2.25)	0.003 (– 0.14 to 0.067)	0.06	0.96	0.03 (– 0.12 to 0.18)	0.07	0.69

The table shows the average change from baseline to post-tcVNS and follow-up for each group with associated standard deviation, estimates for the main effect of treatment and the treatment × Time interaction with standard errors and associated *p* values

^a^Values are reported as average change with corresponding standard deviation

^b^Values are shown as median change with interquartile ranges

**Fig. 7:**
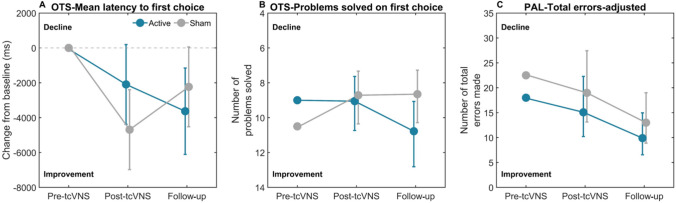
Estimated marginal means (± 95% CI) for the change from baseline (grey dotted line) in the OTS-MLFC (**A**), OTS-PSFC (**B**), and PAL-TEA (**C**) for active and sham tcVNS groups. *ms* milliseconds. Note the OTS-PSFC plot has been inverted with lower values at the top of the figure for consistency

Estimates for the three CANTAB tests measuring executive function and visual memory are shown in Table 5. For this analysis, two participants were excluded; one due to a measurement error (sham group) and another where the participant stopped the baseline assessment prematurely (active group), leaving 15 in the active group and 16 in the sham group. The generalised linear mixed models for OTS-PSFC and PAL-TEA showed no statistically significant main effect of treatment or a treatment × time interaction. For OTS-MLFC, the main effect of treatment (*B* = 2595.9, *p* = 0.13) indicated a trend towards greater improvement in the sham relative to the active group (Fig. 7A) post-tcVNS, although both groups improved. The interaction between treatment and time showed also a trend towards significance (*B* = – 3994.3, *p* = 0.08), suggesting a potential difference in the change from post-tcVNS to follow-up between the groups. The estimated marginal means indicate that while the sham group declines, the active group continues to improve.

**Table 5:** Results from the linear mixed model intention-to-treat analysis comparing active tcVNS with sham tcVNS on the cognitive function of executive function and visual memory. The table shows the average change from baseline to post-tcVNS and follow-up for each group, estimates for the main effect of treatment and the treatment × time interaction with standard errors, and associated p values

Outcome	Active tcVNS, mean (SD)		Sham tcVNS, mean (SD)		Main effect of treatment (at 12 weeks; post-tcVNS)			Treatment × time interaction (change from post-tcVNS [12 weeks] to follow-up [24 weeks])		
	Δpost-tcVNS	Δfollow-up	Δpost-tcVNS	Δfollow-up	Estimate	SE	*p* value	Estimate	SE	*p* value
OTS-MLFC (ms)^a^	– 1142.8 (5853.7)	– 1935.3 (5594.5)	– 5900.2 (9237.2)	– 3443.3 (7072.9)	2595.87 (– 751.5 to 5943.3)	1668.17	0.13	– 3994.32 (– 8481.4 to 492.8)	2236.11	0.08
OTS-PSFC (n)^b^	0 (2.75)	2 (6.5)	0 (3)	0 (3)	0.04 (– 0.24–0.23)	0.12	0.76	0.18 (– 0.15 to 0.52)	0.17	0.29
PAL-TEA (n)^b^	– 6 (17)	– 4 (12.75)	– 1 (13.25)	– 10 (13)	– 0.23 (– 0.79 to 0.33)	0.29	0.42	– 0.04 (– 0.53–0.45)	0.25	0.87

^a^Values are reported as average change with corresponding standard deviation

^b^Values are shown as median change with interquartile ranges

Results from the exploratory ‘per-protocol’ analysis are presented in Supplementary Table 5. This analysis indicated a significant main effect of treatment post-tcVNS (*B* = 3790.0, *p* = 0.047). Participants in the sham group showed greater improvement between baseline and post-tcVNS, although both groups improved. There was also a significant treatment × time interaction for the OTS-MLFC measure (*B* = – 5059.65, *p* = 0.016). Participants in the active group demonstrated greater improvement between post-tcVNS and follow-up relative to the sham group (Supplementary Fig. 2).

## Discussion

In this double-blind, randomised controlled trial, we have demonstrated the feasibility of our protocol, that multi-dose domiciliary tcVNS is acceptable in people with PD and high adherence to the recommended dosing schedule. Importantly, tcVNS was safe in our study, with minimal adverse events thought to be related to the device. However, our data do not demonstrate that multi-dose domiciliary tcVNS improves dopa-resistant gait characteristics or cognitive function of attention, executive function and visual memory in PD.

### Evaluating domiciliary tcVNS in people with Parkinson’s disease

Feasibility was assessed through key indicators, including consent, eligibility, recruitment, and retention rates. Comparing these feasibility metrics across non-pharmacological intervention studies can be challenging due to variations in definitions, recruitment strategies, and the nature of the interventions. Furthermore, comprehensive reporting of feasibility indicators is often lacking, making it difficult to benchmark findings effectively.

Previous studies have explored the feasibility, safety, and preliminary efficacy of tVNS on outcomes such as gait, cognition, and clinical severity in PD [30, 52–56]. However, explicit reporting of primary feasibility metrics specific to tcVNS interventions remains limited. This gap underscores the importance of our study in providing a detailed and systematic evaluation of these indicators, which can serve as a reference for future studies.

Our consent rate was good and aligns closely with the 49% consent rate reported by Best et al., [36]. Also, our eligibility rate can be considered relatively high given our strict exclusion criteria. The primary reasons for screening failure were autonomic dysfunction, including clinically significant hypotension, bradycardia, or tachycardia identified during medical screening. However, this eligibility rate may not be representative of the general patient population as our study exclusively included individuals from the northeast of England who had expressed willingness to participate in research. In comparison, our eligibility rate is somewhat lower than that reported in a similarly sized physical intervention study [57] but comparable to rates observed in another neuromodulation study focusing on improving dual-task gait in PD using transcranial direct current stimulation [58].

The recruitment rate was fairly low, and although we did not reach our planned sample size of 40 participants, we achieved 83% of our target with 33 participants. This aligns with the sample size reported by a recent multi-dose domiciliary tcVNS study in PD [54]. Falling short of recruitment targets is a common challenge in clinical trials; a scoping review of NIHR-funded randomised controlled trials published between 1997 and 2020 found that only 63% of trials recruited to target, while 22% recruited to within 80% of their goal [59].

The calculated retention rates were excellent and exceeded those reported by Mondal and colleagues [54]. The retention rate in our study is also comparable to rates reported in non-stimulation intervention trials using virtual reality or exercise to improve gait and motor function in PD, which range from 92% [60] to 95.7% [61].

### Adherence and acceptability of multi-dose tcVNS in Parkinson’s disease

Adherence to tcVNS was generally high, with most participants in both groups demonstrating full adherence (≥ 80% of expected usage). The percentage of days with zero usage was relatively low overall, but slightly higher in the sham group compared to the active group. This suggests that participants in the active group were more likely to engage with the device consistently. Responses to the usability questionnaire provided additional insights into participants’ perception and satisfaction with the device. This suggested that interventions delivered at home may be more acceptable to patients than clinic-based approaches [52]. The acceptability of tcVNS in PD was assessed by calculating the completion rate over the 12-week intervention period. Approximately a quarter of participants stopped using the device due to potential adverse events. All were, however, consistent with recognised side effects of vagus nerve stimulation [62]. Despite discontinuation of the device, all participants remained in the study as intention to treat.

### The safety of multi-dose tcVNS in people with Parkinson’s disease

tVNS is widely regarded as a safe neurostimulation technique with minimal side effects, the most common being local skin irritation at the electrode site and headache [62, 63]. Serious adverse events associated with tVNS in PD are rare [25].

In our study, few participants reported adverse events during the intervention period, and most were of mild-to-moderate severity, which likely contributed to the high retention rates observed in our trial. Adverse events were reported in both active and sham groups, although the type of events varied between groups, findings that align with prior reports [25]. In our trial, the most frequently reported adverse event was falls. However, as falls are a hallmark motor symptom of PD [64, 65], their occurrence in this study is unlikely to be attributable to tcVNS. Only one participant (in the sham group) experienced their first fall during the intervention period. In addition to falls, two participants, one in each group, reported worsening parkinsonism. Two participants in our study had an unanticipated change in their medications. While these changes were not thought to be related to tcVNS, further investigations are needed to explore potential interactions between tcVNS and PD medications to better understand any underlying mechanisms.

During the follow-up period after stimulation had been completed, fewer adverse events were reported by participants, and none were considered related to prior tcVNS use. This may be attributed to the reduction in contact with the study team after the intervention period. There were, however, five serious adverse events in the same participant during two separate hospital admissions, which were unrelated to the study device.

### The efficacy of tcVNS on gait and cognition in Parkinson’s

A secondary aim of this study was to evaluate the efficacy of tcVNS on discrete gait characteristics and cognitive domains, including attention, executive function, and visual memory. To date, this is the longest tcVNS intervention study in PD, with a 12-week domiciliary protocol. Previous research has primarily focused on shorter interventions (1 month) or single-dose designs, including assessments of axial motor symptoms [54] and cognitive effects [53].

Our findings were not in line with our hypotheses and suggest minimal effects of multi-dose domiciliary tcVNS on dopa-resistant gait characteristics, which is in contrast with other studies in PD reporting improvements in discrete gait characteristics including stride length, swing amplitude, gait speed, and step length following tVNS [53–55]. Nonetheless, we observed a small, non-significant reduction of 0.56 cm in step length variability in the active group post-tcVNS compared to the sham group. While reduced gait variability is generally associated with a lower risk of falls in PD [66], we did not observe a corresponding reduction in fall frequency in our study. This suggests that any potential effects of tcVNS on gait stability were modest, short-lived, and not sufficient to influence clinical outcomes. Indeed, any observed improvements in gait variability had dissipated by the 12-week follow-up, returning to baseline levels.

In addition to gait, we assessed the effects of tcVNS on cognitive function but found no significant effects of the intervention on assessments measuring attention, executive function, and visual memory. Marginal improvements in spatial planning (measured via the OTS task) were observed in the active group, with further gains at follow-up, but these did not differ significantly from the sham group. Exploratory ‘per-protocol’ analysis demonstrated that both groups improved on this task post-tcVNS and that reaction times significantly improved in the active group relative to the sham group at follow-up. Improvements in both groups could feasibly be associated with placebo effects of the intervention.

The effects of tVNS on cognitive function have been widely studied (see comprehensive reviews [67, 68]), with some evidence suggesting cognitive benefits across various populations [67]. Our findings align with studies in PD that report no significant cognitive effects of tVNS [52], though others have observed improvements in response times following a single tVNS dose [53].

### Unresolved issues for tVNS in clinical trials: location, lateralisation, and optimal period of stimulation

Considerable variability exists across studies of tVNS due to differences in study design and stimulation protocols. For instance, the mechanisms underlying auricular versus cervical vagus nerve stimulation remain poorly understood. While auricular stimulation likely activates only afferent vagal fibers [17, 68], cervical stimulation engages both afferent and efferent fibers, potentially eliciting broader effects [69]. Head-to-head comparisons are needed to elucidate these differences.

Lateralisation is another unresolved issue. In this study, we stimulated the left vagus nerve, as is standard practice for auricular and cervical tVNS [62, 68]. However, emerging evidence suggests that right-sided stimulation may have a comparable safety profile [70, 71]. Whether left and right stimulation elicits differential effects is an understudied topic.

The optimal stimulation period for PD remains unclear. Our pilot study showed significant effects of a single tcVNS dose on gait characteristics associated with cholinergic neurotransmission [30], but minimal effects were observed in this trial following a 12-week multi-dose regimen. Several factors may contribute to this discrepancy. For example, although cholinergic denervation occurs early in PD [11, 72], it may not have progressed sufficiently in our participants for them to fully benefit from the stimulation. A more granular, personalised approach that considers clinical subtypes of PD, such as brain-first vs. body-first presentations [73], may help identify potential responders and non-responders. Additionally, longer intervention periods could clarify whether repeated, multi-dose tcVNS amplifies the effects observed after a single dose, as chronic stimulation has shown cumulative benefits in other conditions such as depression [74].

### Study strengths and limitations

The primary aim of this study was not to evaluate the definitive effects of tcVNS on key motor and non-motor symptoms of PD. Instead, proof-of-concept studies like ours are designed to provide important information to optimise trial procedures and outcome measures in preparation for larger-scale trials [34]. For example, the standard deviation of change in measures observed in this study provides a useful estimate for modelling the required sample size to detect possible clinically meaningful effects of tcVNS on, for example, discrete gait characteristics. Here, we report findings from the longest intervention study of multi-dose domiciliary tcVNS in PD. A notable strength of this trial is the high retention rate across both active and sham groups, demonstrating strong adherence to and acceptability of tcVNS among participants. Additionally, this study highlights the safety of tcVNS in people with PD, with no serious adverse events during the treatment period. Our sample size was smaller than originally planned of 40 participants, likely due to the stringent exclusion criteria necessary, given the lack of prior safety data for tcVNS in PD. Consequently, our findings may not be generalisable to the broader PD population and may have also obscured potential statistically significant effects of tcVNS on our secondary outcomes. Finally, participants in the sham group exhibited higher LEDD, which may have influenced our findings regarding the efficacy of tcVNS on gait and cognition. Although LEDD was incorporated as a covariate in all statistical models, it remains uncertain whether dopaminergic replacement therapies affect the response to tVNS. Moreover, residual confounding by Hoehn and Yahr stage cannot be excluded given the small sample and subgroup imbalance. Future studies may consider balancing LEDD and Hoehn and Yahr staging during randomisation.

## Conclusions

Overall, this study successfully establishes the feasibility of multi-dose tcVNS as a novel therapeutic approach in PD. We furthermore demonstrate that tcVNS as an adjunctive intervention in people with PD is acceptable and safe. Even though we demonstrate limited effects of multi-dose tcVNS on gait variability and cognitive function, this study has important implications for future studies that wish to assess the efficacy of tcVNS on key motor and non-motor symptoms in PD.

## Supplementary Information

Below is the link to the electronic supplementary material.

Supplementary file1 (DOCX 194 KB)

## Data Availability

Data will be made available to academic researchers following a reasonable request.
