# Supplementary material for: Feasibility, safety and efficacy of multi-dose vagus nerve stimulation in Parkinson’s disease: a double-blind, randomised sham-controlled proof-of-concept study
Source: J Neurol. 2025 Oct 9;272(10):684. doi: 10.1007/s00415-025-13430-4 (PMC12511173; doi:10.1007/s00415-025-13430-4)
Supplement: Supplementary file 1 — Supplementary file1 (DOCX 194 KB) [file 415_2025_13430_MOESM1_ESM.docx]

Supplementary material to:

**Feasibility, Safety and Efficacy of Multi-Dose Vagus Nerve Stimulation in Parkinson’s Disease: a Double-blind, Randomised Sham-controlled Proof-of-Concept Study**

*Hilmar P. Sigurdsson, Heather Hunter, Lisa Alcock, Evangeline E Maughan, Harvey Bramley, Philip Brown, Giovanni Palermo, Mark R. Baker, John-Paul Taylor, Lynn Rochester & Alison J. Yarnall*

Correspondence: Alison J. Yarnall <alison.yarnall@newcastle.ac.uk>

# Methodology

## Protocol amendments

The eligibility criteria were modified from an age range of 18–75 years to 18-<76 years, expanding eligibility to include women of child-bearing potential provided they agreed to use two effective forms of contraception throughout the study duration. Additionally, the exclusion criteria were adjusted to permit participants with a history of non-metastatic cancers that had been appropriately treated, specifically non-melanoma skin carcinoma and localised prostate cancer. To accommodate participant availability and laboratory scheduling, we also amended the window for follow-up visits. The post-tcVNS and follow-up assessments, originally scheduled within 7 days (5 working days) of the 12-week periods, were adjusted to a flexible window of -3 to +7 days to accommodate scheduling constraints.

## Primary outcomes definitions

*Feasibility*

*Consent rate*: Calculated as the proportion of participants who provided informed consent out of those initially approached with study information.

*Eligibility rate*: Proportion of participants who met the study criteria out of those who provided informed consent and proceeded to recruitment and randomisation. This metric reflects the generalisability of the study population and therefore the intervention for the target population [1]

*Recruitment rate*: Calculated as the ratio of the number of participants recruited and randomised to the total number approached and provided with study information.

*Retention rate*: Calculated as the proportion of participants included in the primary outcome analyses out of those randomised. A differential retention rate was also computed by group.

*Adherence*

Adherence was evaluated using device-recorded usage data extracted at the end of the study and calculated, for each participant, as the ratio of total minutes of active device use to the total minutes expected over the 12-week intervention (i.e., 4 stimulations per day times 120 seconds). Participants were categorised into three predefined adherence groups [2]: *Full adherence* (≥80% of expected usage), *partial adherence* (≥50%–<80%), and *non-adherence* (<50%). Additionally, we calculated the percentage of non-use days (i.e., days with zero recorded usage). For participants who withdrew or stopped using the device prematurely, adherence was calculated based on data from baseline to their final day of use.

*Acceptability*

*Completion rate:* Defined as the proportion of participants who continued using the device throughout the 12-week intervention period out of those who were initially recruited and randomised. *Intervention satisfaction*: To assess the perception, satisfaction, and ease of use of tcVNS in PD, we used a modified version of the usability questionnaire by Rabinovich and colleagues [3]. This modified version included items on a 5-point ordinal scale, with 5 being the most favourable and 1 being the least favourable option. For this analysis, responses from both groups were pooled.

*Safety*

Safety was monitored by recording adverse events reported by participants throughout the study period. We defined adverse events as any unfavourable and unintended sign, symptom, syndrome or illness that develops or worsens during the period of observation in the study. Adverse events were classified as possibly related or unrelated to tcVNS based on their temporal association with device use and the timing of sign, symptom, syndrome or illness onset, plus knowledge of the known side effect profile.

## Secondary outcomes

*Gait assessment*

Supplementary figure 1 illustrates the 25 m oval circuit used for all gait assessments across participants and sessions. The circuit incorporated two wide, arched turns—one at each end—to avoid abrupt changes in direction (e.g., turns). The instrumented gait mat was located on a straight section of the circuit, with approximately 1.5 m of straight walkway before and after the mat to account for acceleration into, and deceleration out of, the arched turns.


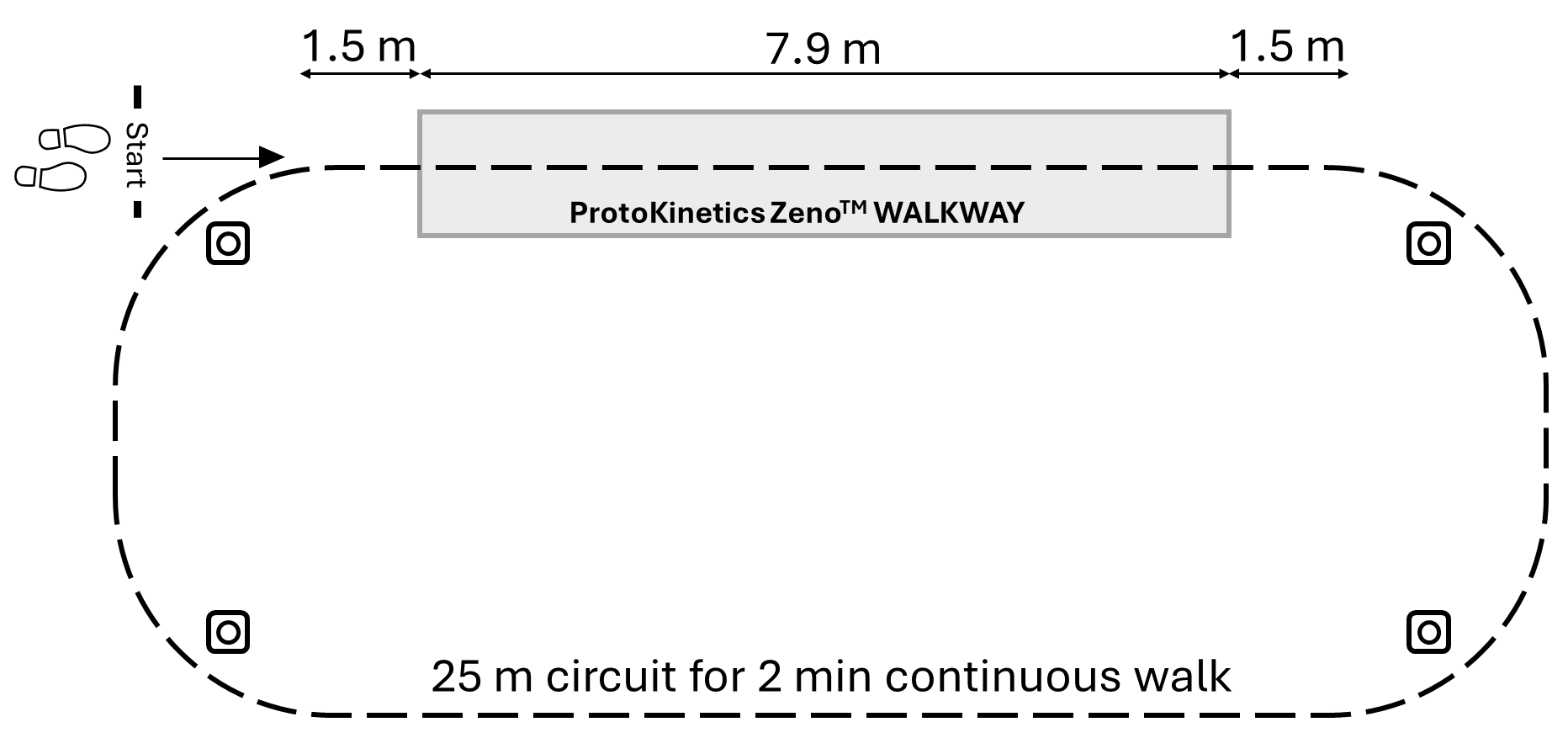


**Supplementary figure 1.** A schematic diagram of the circuit used for all gait assessments in the study.

# Results

## Adverse events over the 12-week follow-up period

Eight (50.0%) participants in the active group and seven (46.7%) participants in the sham group reported adverse events during the 12-week non-stimulation follow-up period (i.e., not during the active stimulation period; see Supplementary Table 1 for details). Five serious adverse events were reported in the same participant over a short period of time when hospitalised on two separate occasions and classified as unrelated to the study device. These were acute kidney injury secondary to acute urinary retention and infection, with subsequent pre-syncope due to medication prescribed for urinary retention. The same participant developed small bowel obstruction, which settled spontaneously. The participant recovered and was seen again at their 24-week follow-up. This participant was in the active group. A review of this participant’s device usage showed minimal adherence to the dosing schedule, and they were classified in the non-adherence group.

**Supplementary Table 1.** Adverse events reported by participants in the active and sham groups over the 12-week non-stimulation, follow-up period.

|  | Active tcVNS (n=16) | | Sham tcVNS (n=15) | |
| --- | --- | --- | --- | --- |
|  | Number of participants (%) | Number of Events | Number of participants (%) | Number of Events |
| Acute kidney injury | 1 (6.3%) | 1 | 0 (0.0%) | 0 |
| Acute urinary retention | 1 (6.3%) | 1 | 0 (0.0%) | 0 |
| Cardiovascular symptoms*^a^* | 0 (0.0%) | 0 | 2 (13.3%) | 3 |
| Fall | 1 (6.3%) | 1 | 4 (26.7%) | 4 |
| Fatigue | 1 (6.3%) | 1 | 0 (0.0%) | 0 |
| Foot dragging | 0 (0.0%) | 0 | 1 (6.7%) | 2 |
| Gastrointestinal disturbance*^b^* | 0 (0.0%) | 0 | 1 (6.7%) | 1 |
| Infection*^c^* | 1 (6.3%) | 1 | 2 (13.3%) | 2 |
| Pain*^d^* | 3 (18.8%) | 3 | 0 (0.0%) | 0 |
| Pre-syncope | 1 (6.3%) | 1 | 0 (0.0%) | 0 |
| Small bowel obstruction | 1 (6.3%) | 1 | 0 (0.0%) | 0 |
| Urinary urgency | 1 (6.3%) | 1 | 0 (0.0%) | 0 |
| *Note: Two participants in the sham group withdrew during the intervention period. ^a^Cardiovascular symptoms included orthostatic hypotension (n=2). ^b^Gastrointestinal disturbance included constipation (n=1). ^c^Infection included COVID-19 infection (n=2), lower respiratory tract infection (n=1) and urinary tract infection (n=1). ^d^Pain included abdominal pain (n=2) and lower knee pain (n=1).* | | | | |

## Dopa-resistant gait outcomes

**Supplementary table 2** presents raw means and standard deviations for all dopa-resistant gait characteristics analysed in the study assessed in active and sham groups at baseline, post-tcVNS and follow-up. These data are provided to complement the model-based estimates along with mean change (Δ) scores and associated standard deviations reported in the main text.

**Supplementary table 2**. Raw means and standard deviations for active and sham groups in the intention-to-treat sample on dopa-resistant gait characteristics along with average change from baseline to post-tcVNS and follow-up for each group.

| Variable (units) | Group | n timepoints | Baseline | Post tcVNS | Follow-up |
| --- | --- | --- | --- | --- | --- |
| Step length variability (cm) | Active | 15 / 14 / 13 | 2.71(± 1.28) | 1.92(± 0.78) | 2.54(± 1.50) |
|  | Sham | 16 / 14 / 14 | 2.22(± 0.74) | 2.21(± 0.84) | 1.79(± 0.81) |
| Step time variability (s) | Active | 15 / 14 / 13 | 0.025(± 0.012) | 0.021(± 0.004) | 0.028(± 0.021) |
|  | Sham | 16 / 14 / 14 | 0.021(± 0.01) | 0.020(± 0.004) | 0.021(± 0.007) |

## ‘Per-protocol’ analyses of step length and step time variability

**Supplementary Table 3**. A per-protocol analysis comparing active tcVNS (n = 10) with sham tcVNS (n = 12) on the variability of step length and step time. The table presents the average change from baseline to post-tcVNS and follow-up for each group, along with the corresponding standard deviation, main effect, interaction estimates, standard errors, and p-values.

|  | Main effect of Treatment (after 12-weeks; post-tcVNS). | | | Treatment x Time interaction (change from post-tcVNS [12-weeks] to follow-up [24-weeks]) | | |
| --- | --- | --- | --- | --- | --- | --- |
| Outcome | Estimate  (95% CI) | SE | *p* value | Estimate | SE | *p* value |
| Step length variability (cm) | -0.66  (-1.55 – 0.23) | 0.438 | 0.14 | 0.979  (-0.16 – 2.12) | 0.547 | 0.09 |
| Step time variability (s) | -0.0007  (-0.01 – 0.01) | 0.006 | 0.889 | 0.005  (-0.009 – 0.02) | 0.007 | 0.45 |

## ‘Per-protocol’ analyses on cognitive measures of attention

**Supplementary Table 4**. A per-protocol analysis comparing active tcVNS (n = 10) with sham tcVNS (n = 12) on measures of attention. The table presents the average change from baseline to post-tcVNS and follow-up for each group, along with the corresponding standard deviation, main effect, interaction estimates, standard errors, and p-values.

|  | Main effect of Treatment (at 12-weeks; post-tcVNS). | | | Treatment x Time interaction (change from post-tcVNS [12-weeks] to follow-up [24-weeks]) | | |
| --- | --- | --- | --- | --- | --- | --- |
| Outcome | Estimate  (95% CI) | SE | *p* value | Estimate | SE | *p* value |
| PoA (ms) | 37.33  (-63.13 – 137.78) | 49.02 | 0.45 | -67.32  (-164.2 – 29.55) | 46.48 | 0.16 |
| FA (%) | 10.47  (-6.87 – 27.81) | 8.51 | 0.23 | -5.71  (-24.58 – 13.16) | 9.07 | 0.54 |
| PS (ms) | 9.67  (-30.77 – 50.11) | 19.87 | 0.63 | -10.90  (-56.29 – 34.48) | 21.82 | 0.62 |
| CoA (n)^a^ | 0.014  (-0.10 – 0.13) | 0.06 | 0.82 | 0.02  (-0.13 – 0.16) | 0.08 | 0.83 |

## ‘Per-protocol’ analyses on executive function and visual memory

**Supplementary Table 5.** A per-protocol analysis comparing active tcVNS (n = 10) with sham tcVNS (n = 12) on measures of executive function and visual memory. The table presents the average change from baseline to post-tcVNS and follow-up for each group, along with the corresponding standard deviation, main effect, interaction estimates, standard errors, and p-values.

| Outcome | Main effect of Treatment (at 12-weeks; post-tcVNS). | | | Treatment x Time interaction (change from post-tcVNS [12-weeks] to follow-up [24-weeks]) | | |
| --- | --- | --- | --- | --- | --- | --- |
|  | Estimate  (95% CI) | SE | *p* value | Estimate | SE | *p* value |
| OTS-MLFC (ms) | 3790  (46.37 – 7533.7) | 1845.2 | **0.047** | -5745.1  (-10281 – -1208.8) | 2181.3 | **0.016** |
| OTS-PSFC (n) | -0.02  (-0.32 – 0.27) | 0.15 | 0.89 | 0.30  (-0.09 – 0.68) | 0.20 | 0.13 |
| PAL-TEA (n) | -0.08  (-0.67 – 0.52) | 0.30 | 0.80 | 0.08  (-0.45 – 0.62) | 0.27 | 0.77 |


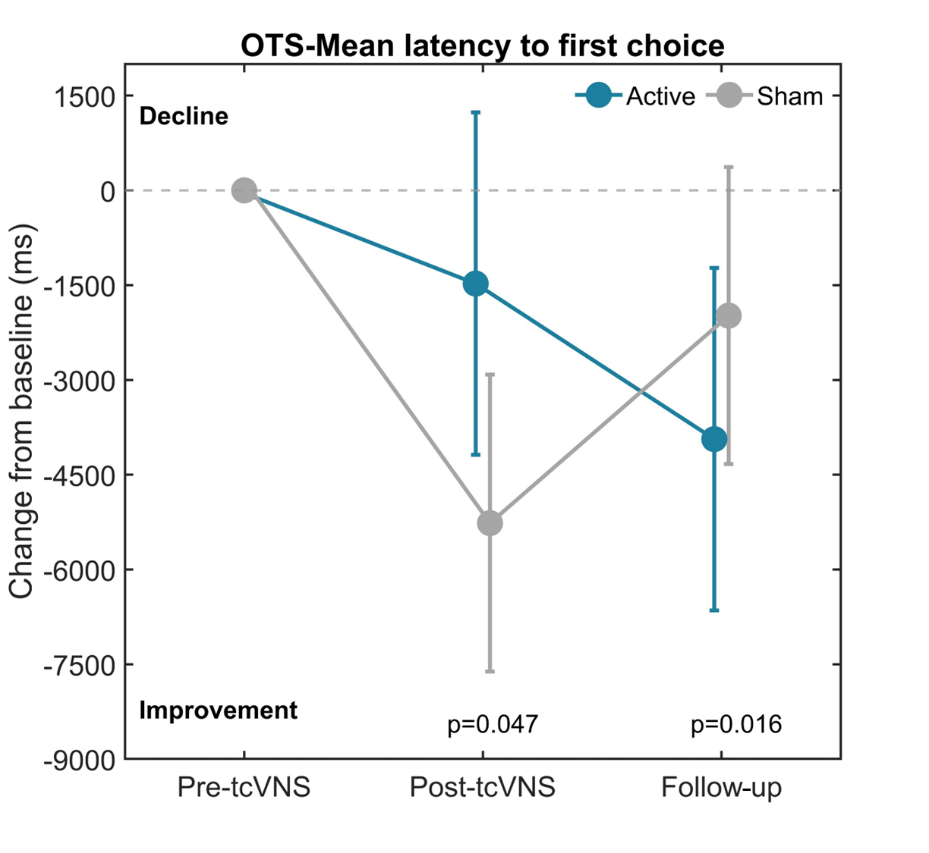


**Supplementary Figure 2.** Estimated marginal means (± 95% CI) for the change from baseline (dotted grey line) in OTS-MLFC for the Active and Sham tcVNS groups in the exploratory per-protocol analysis. Positive values indicate increased response times (decline) whereas negative values indicate reduced response time (improvement). Abbreviation: ms – milliseconds.

# Supplementary material references

1. Hubbard G, O’Carroll R, Munro J, Mutrie N, Haw S, Mason H, Treweek S (2016) The feasibility and acceptability of trial procedures for a pragmatic randomised controlled trial of a structured physical activity intervention for people diagnosed with colorectal cancer: findings from a pilot trial of cardiac rehabilitation versus usual care (no rehabilitation) with an embedded qualitative study. Pilot Feasible Stud 2(1):1-15. <https://doi.org/10.1186/s40814-016-0090-y>

2. Conraads VM, Deaton C, Piotrowicz E, Santaularia N, Tierney S, Piepoli MF, . . . Jaarsma T (2012) Adherence of heart failure patients to exercise: barriers and possible solutions: a position statement of the Study Group on Exercise Training in Heart Failure of the Heart Failure Association of the European Society of Cardiology. Eur J Heart Fail 14(5):451-458. <https://doi.org/10.1093/eurjhf/hfs048>

3. Rabinovich RA, Louvaris Z, Raste Y, Langer D, Van Remoortel H, Giavedoni S, . . . Consortium P (2013) Validity of physical activity monitors during daily life in patients with COPD. European Respiratory Journal 42(5):1205-1215. <https://doi.org/10.1183/09031936.00134312>
